# Synthesis, Physical Properties and Enzymatic Degradation of Biodegradable Nanocomposites Fabricated Using Poly(Butylene Carbonate-Co-Terephthalate) and Organically Modified Layered Zinc Phenylphosphonate

**DOI:** 10.3390/polym12092149

**Published:** 2020-09-21

**Authors:** Li-Ying Tseng, Erh-Chiang Chen, Jie-Mao Wang, Tzong-Ming Wu

**Affiliations:** Department of Materials Science and Engineering, National Chung Hsing University, 250 Kuo Kuang Road, Taichung 402, Taiwan; tzeng0808@gmail.com (L.-Y.T.); erchiang.chen@gmail.com (E.-C.C.); D9866023@mail.nchu.edu.tw (J.-M.W.)

**Keywords:** biodegradable copolyesters, composites, mechanical property, enzymatic degradation

## Abstract

A new biodegradable aliphatic-aromatic poly (butylene carbonate-co-terephthalate) (PBCT-85) with the molar ratio [BC]/[BT] = 85/15, successfully synthesized through transesterification and polycondensation processes, was identified using 1H-NMR spectra. Various weight ratios of PBCT/organically modified layered zinc phenylphosphonate (m-PPZn) nanocomposites were manufactured using the solution mixing process. Wide-angle X-ray diffraction and transmission electron microscopy were used to examine the morphology of PBCT-85/m-PPZn nanocomposites. Both results exhibited that the stacking layers of m-PPZn were intercalated into the PBCT-85 polymer matrix. The additional m-PPZn into PBCT-85 copolymer matrix significantly enhanced the storage modulus at −70 °C, as compared to that of neat PBCT-85. The lipase from *Pseudomonas sp.* was used to investigate the enzymatic degradation of PBCT-85/m-PPZn nanocomposites. The weight loss decreased as the loading of m-PPZn increased, indicating that the existence of m-PPZn inhibits the degradation of the PBCT-85 copolymers. This result might be attributed to the higher degree of contact angle for PBCT-85/m-PPZn nanocomposites. The PBCT-85/m-PPZn composites approved by MTT assay are appropriate for cell growth and might have potential in the application of biomedical materials.

## 1. Introduction

Aliphatic polycarbonates (APCs) prepared by copolymerization of CO2 and epoxides or the ring-opening polymerization method have attracted lots of interest as biodegradable polymers, due to their biocompatibility, biodegradability, and nontoxicity [1,2,3,4]. Among the obtaining APCs, poly (butylene carbonate) (PBC) has received significant attention owing to its promising comprehensive properties and competitive cost [5,6]. Nevertheless, the low melting temperature and a slow crystallization rate of PBC could restrict its application. In accordance with previous studies, the incorporation of aromatic ester monomer into PBC backbone to synthesize the poly(carbonate-co-ester)s with random chain conformation could change the physical properties of PBC [7,8]. A similar approach was applied to the preparation of commercially available product poly(butylene adipate-co-terephthalate) (PBAT, Ecoflex^®^, BASF), as compared to poly (butylene adipate) (PBA). It was reported that the crystallization behavior of PBA could be enhanced by copolymerizing with aromatic polyesters [9].

Consequently, this study intends to synthesize poly (butylene carbonate-co-terephthalate) (PBCT) copolymer with suitable properties via transesterification and the polycondensation method. In order to further enhance these mechanical and thermal properties of PBCT copolymer, the addition of excellent physical properties of inorganic materials acting as nucleating agents and reinforcements into the polymer matrix is undertaken to accelerate their crystallization rates and improve their physical properties [10,11,12,13]. Reinforcing layered zinc phenylphosphonates (PPZn) has received significant attention owing to the enhancement of the crystallization rates and physical properties of polymers [9,10,14,15,16]. The interlayered spacing of PPZn is too small to fabricate the exfoliated or intercalated polymer nanocomposites. Organo-modifiers with functional group are necessary to enlarge the interlayered spacing of PPZn.

In this study, two biocompatible dodecylamine (DDA) and octadecylamine (ODA)—long-chain primary alkylamines—were used in this work to fabricate the organically-modified PPZn (m-PPZn) through the anion exchange method. A series of new poly (butylene carbonate-co-terephthalate)/organically modified layered zinc phenylphosphonates nanocomposites were successfully synthesized. To our knowledge, no report on PBCT/PPZn or PBCT/m-PPZn nanocomposite has been discussed. The mechanical, thermal and enzymatic degradation properties of PBCT/m-PPZn nanocomposites were studied systematically. Finally, the potential of the PBCT/m-PPZn composites as biomedical materials was investigated using a 3-(4,5-dimethylthiazol-2-yl)-2,5-diphenyltetrazolium bromide (MTT) assay with L929 fibroblast cells.

## 2. Materials and Methods

### 2.1. Materials

Lipase from *Pseudomonas sp.*, dodecylamine (DDA), octadecylamine (ODA), zinc nitrate, and phenylphosphonic acid was acquired from Sigma-Aldrich Chemical Company (St. Louis, MO, USA). 1,4-butanediol (BD), dimethylene carbonate (DMC), and dimethylene terephthalate (DMT) were obtained from Alfa Aesar Chemical Company (Haverhill, MA, USA). Sodium hydroxide was purchased from Fluka Chemical Company (Buchs, Switzerland). All chemicals were used without purification.

### 2.2. Fabrication of PBCT/m-PPZn Nanocomposites

The PBCT with molar ratio of butylene carbonate (BC) to butylene terephthalate (BT) was 85:15, which was synthesized via transesterification and polycondensation; the resulting product is hereinafter designated as PBCT-85. In brief, desirable amounts of DMC, DMT, BD, and sodium hydroxide as a catalyst were heated and mechanically stirred at 120 °C for 1 h in a stream of nitrogen gas, and then heated to 190 °C for 1 h, and finally heated to 220 °C for 4 h in a vacuum of 67 Pa. For purification of PBCT, the as-prepared PBCT was dissolved in 200 mL dichloromethane and then precipitated from 2 L cold methanol. The above purification process needs to be repeated three times. The PPZn and organically modified PPZn (m-PPZn) with long-chain primary alkylamine, such as dodecylamine (DDA) and octadecylamine (ODA), were fabricated using the approach reported in previous literatures [17,18]. Different amounts of PBCT-85 and m-PPZn were individually dissolved in dichloromethane, and then mixed/mechanically stirred for 3 days. The obtained 1, 3, and 5 wt% PBCT-85/m-PPZn nanocomposites were washed and dried in vacuum.

### 2.3. Analytical Procedures

Experimental measurements of wide-angle X-ray diffraction (WAXD) and small-angle X-ray scattering (SAXS) were performed using an X-ray diffractometer (Bruker D8, Bruker, Billerica, MA, USA) equipped with a Ni-filtered Cu Kα radiation source. In WAXD measurement, the diffraction patterns were carried out in the range of 2*θ* = 1.5°–30° at a scanning rate of 1°/min. In situ WAXD experiments were recorded under vacuum using a temperature-attachment assembly. The increasing rate of applied temperature was 10 °C/min and the applied temperature was stabilized for 5 min before each measurement. The degree of crystallinity was calculated by WAXD data. In SAXS measurement, the *q* is the scattering vector defined as *q* = (4*π*sin*θ*)/*λ*, where *λ* is he X-ray wavelength. The sample to detector distance is ~300 mm. The transmission electron microscopy (TEM) was performed using Hitachi HF-2000 (Hitachi, Tokyo, Japan). The samples of TEM experiments encapsulated by epoxy were prepared using a Reichert Ultracut ultramicrotome (Reichert, Manila, Philippines). The field-emission scanning electron microscopy (FESEM) conducted at 3 kV using a JEOL JSM-6700F (Tokyo, Japan) field-emission instrument was used to characterize the morphology of the nanocomposites. Fourier transform infrared (FTIR) experiments were carried out on a Perkin–Elmer Spectrum One (Perkin–Elmer, Waltham, MA, USA) spectrometer in the range of 400 to 4000 cm^−1^.

The crystallization behavior was carried out by a PerkinElmer Pyris Diamond DSC (Perkin–Elmer, Waltham, MA, USA), and all measurements were carried out under nitrogen environment. All specimens were heated to the designed temperatures (T_ds_) at a rate of 10 °C/min—about 30 °C higher than the melting temperatures of PBCT-85—and held for 5 min to eliminate the residual crystals. Subsequently, they were cooled to −50 °C at a rate of 10 °C/min. Finally, the samples were heated to T_ds_ at a rate of 10 °C/min and the crystalline melting temperature (T_m_) for the PBCT-85 and PBCT-85/m-PPZn nanocomposites was obtained. The thermal behaviors of samples were operated using Perkin Elmer TG/DTA 6300 thermoanalyzer (Perkin–Elmer, Waltham, MA, USA). These measurements were obtained from room temperature to 800 °C at a heating rate of 10 °C/min under a nitrogen environment.

^1^H-nuclear magnetic resonance (NMR) and ^13^C-NMR spectra were obtained with Agilet Technologies DD2 600MHz NMR (Agilet Technologies, Santa Clara, CA, USA) spectrometer using CDCl_3_ as solvent and internal standard. The gel permeation chromatography (GPC; Waters 717 Plusautosampler, Waters Instruments, Rochester, NY, USA) was used to verify the number-average molecular weight (*M_n_*), weight-average molecular weight (*M_w_*), and polydispersity PDI = *M_w_/M_n_* of the resulting polymers and composite materials. Polystyrene standards with narrow molecular-weight distributions were used as calibration. The storage modulus (E’) was operated on a Perkin Elmer dynamic mechanical analyzer (DMA) (Perkin–Elmer, Waltham, MA, USA) from −80 to 120 °C at 2 °C/min heating rate and 1 Hz constant frequency.

The PBCT-85/m-PPZn nanocomposites were hot pressed at a temperature about 50 °C higher than its melting temperature to prepare the samples for enzymatic degradation analysis. All samples (10 mm × 10 mm) for enzymatic degradation test were put in 24-well plates, including 1 mL/mg lipase from *Pseudomonas sp.* The degraded samples were removed at 3, 6, 9, and 12 days, washed with distilled water, and vacuum-dried. The quantity of degradation was estimated by means of the equation *W_weight loss_* (%) = 100 [(*W_0_* − *W_t_*)/*W_0_*], where *W_0_* is the original weight of a sample and *W_t_* corresponds to the weight of a sample after various degradation periods. The experimental data obtained here are the average values of at least three experiments.

L929 (BCRC RM60091, Hsinchu, Taiwan) cells were incubated on the specimens for 12, 24, and 48 h. Cell suspensions were promptly seeded over each of the specimens in a 24-well plate. After the determined L929-cell incubation time, the cell growth was assayed using the MTT (3-(4,5-dimethylthiazol-2-yl)-2,5-diphenyltetrazolium bromide; Sigma-Aldrich, St. Louis, MO, USA) assay, in which tetrazolium salt is decreased to formazan crystals by the mitochondrial dehydrogenase of living cells. Briefly, 3 h before the end of the incubation time, 20 μL of MTT solution and 180 μL of Dulbecco’s modified Eagle medium (DMEM; Gibco, Langley, OK, USA) containing 1% penicillin/streptomycin were added to each well. Upon removal of the MTT solution, 200 μL of dimethylsulfoxide (DMSO; Sigma-Aldrich, St. Louis, MO, USA) was also added to each well. The plates were then shaken until the formazan crystals had dissolved, and 150 μL of the solution from each well was transferred to a new 96-well plate. Plates were read using a BioTek Epoch spectrophotometer (BioTek, Winooski, VT, USA) at 563 nm. The absorbance results were verified for three independent measurements.

## 3. Results

### 3.1. Synthesis, Structure and Morphology of PBCT-85/m-PPZn Nanocomposites

**Figure 1:**
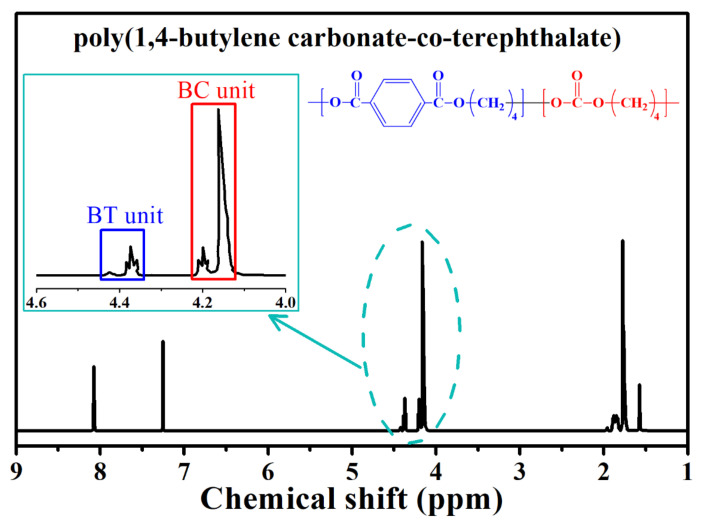
^1^H-nuclear magnetic resonance (H-NMR) spectra of the poly (butylene carbonate-co-terephthalate) (PBCT-85) copolyester.

The chemical composition of PBCT-85 copolymer identified by ^1^H-NMR spectroscopy in CDCl_3_ is shown in Figure 1. Two signals at *δ* = 8.06 and 7.24 ppm are assigned to the phenylene (-C_6_H_4_-) and CDCl_3_ signal. The signals at 4.41 and 4.36 ppm correspond to hydrogen proton of -CH_2_O(CO)C_6_H_4_- for the ester group, and the other signals at 4.19 and 4.13 ppm correspond to hydrogen proton of -CH_2_O(CO)O- for the carbonate group [7,8]. The chemical composition of PBCT-85 copolymer determined using the peak area ratio at *δ* = 4.41–4.36 ppm to that at *δ* = 4.19–4.13 ppm is shown in Table 1. The experimental ratio of carbonate group to ester group is approximately consistent with the feed ratio of [BC] to [BT], implying that the composition of the synthesized PBCT-85 is in good agreement with that calculated approximately on the basis of the feed ratio. The weight average molecular weights and (*M_w_*) and polydisperse index (PDI) of PBCT-85 copolymer established via GPC are 38,110 g/mol and 1.89, respectively. The melting temperature of PBCT-85 established via DSC is 33.7 °C.

**Figure 2:**
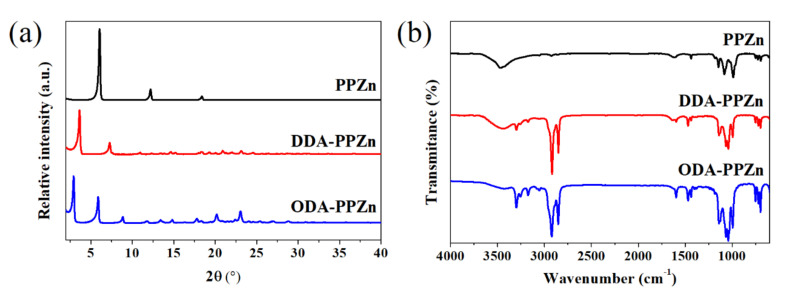
(**a**) X-ray diffraction patterns for PPZn, dodecylamine (DDA)-PPZn and octadecylamine (ODA)-PPZn. (**b**) FTIR spectra of PPZn, DDA-PPZn, and ODA-PPZn.

The X-ray diffraction patterns of PPZn, DDA-PPZn, and ODA-PPZn are presented in Figure 2a. A very strong diffraction of PPZn is observed, revealing the formation of classically and extremely stacked lamellae structure. The adjacent distance of zinc phenylphosphonate layers for PPZn determined using the Bragg’s equation was 14.6 Å. The XRD data of organically-modified PPZn show that the main diffraction peaks are moved to lower angle with the incorporation of dodecylamine and octadecylamine. The interlayer spacings were enlarged to 24.2 and 30.0 Å after the anionic exchange of DDA and ODA, respectively. Figure 2b shows the FTIR spectra of PPZn, DDA-PPZn, and ODA-PPZn. The FT-IR data of PPZn show two absorption peaks at 3465 and 1640 cm^−1^, which are attributed to the stretching mode of monohydrates molecules in the PPZn interlayer gallery. The absorption peaks from 750 to 650 cm^−1^ are assigned to the C=C bands and out-of-plane bands of phenyl. The absorption peaks from 1200 to 950 cm^−1^ are characteristic of PO_3_ group of phosphonic acid. In accordance with the above-mentioned results, these absorption peaks are affiliated with the typical FT-IR spectra of PPZn [17]. The FT-IR spectra of DDA-PPZn and ODA-PPZn present two strong absorption peaks at 2920 and 2860 cm^−1^, which are assigned to the stretching vibration of C–H and =C–H for the alkylamine group in DDA and ODA. The absorption peaks at 1465 and 1070 cm^−1^ contribute to the C–H and C–N bendings of alkylamine group in DDA and ODA. The N–H bending vibration is observed at 1600 cm^−1^. According to the XRD and FT-IR results, DDA and ODA were effectively intercalated into the gallery of PPZn, hence expanding the interlayer spacing.

**Figure 3:**
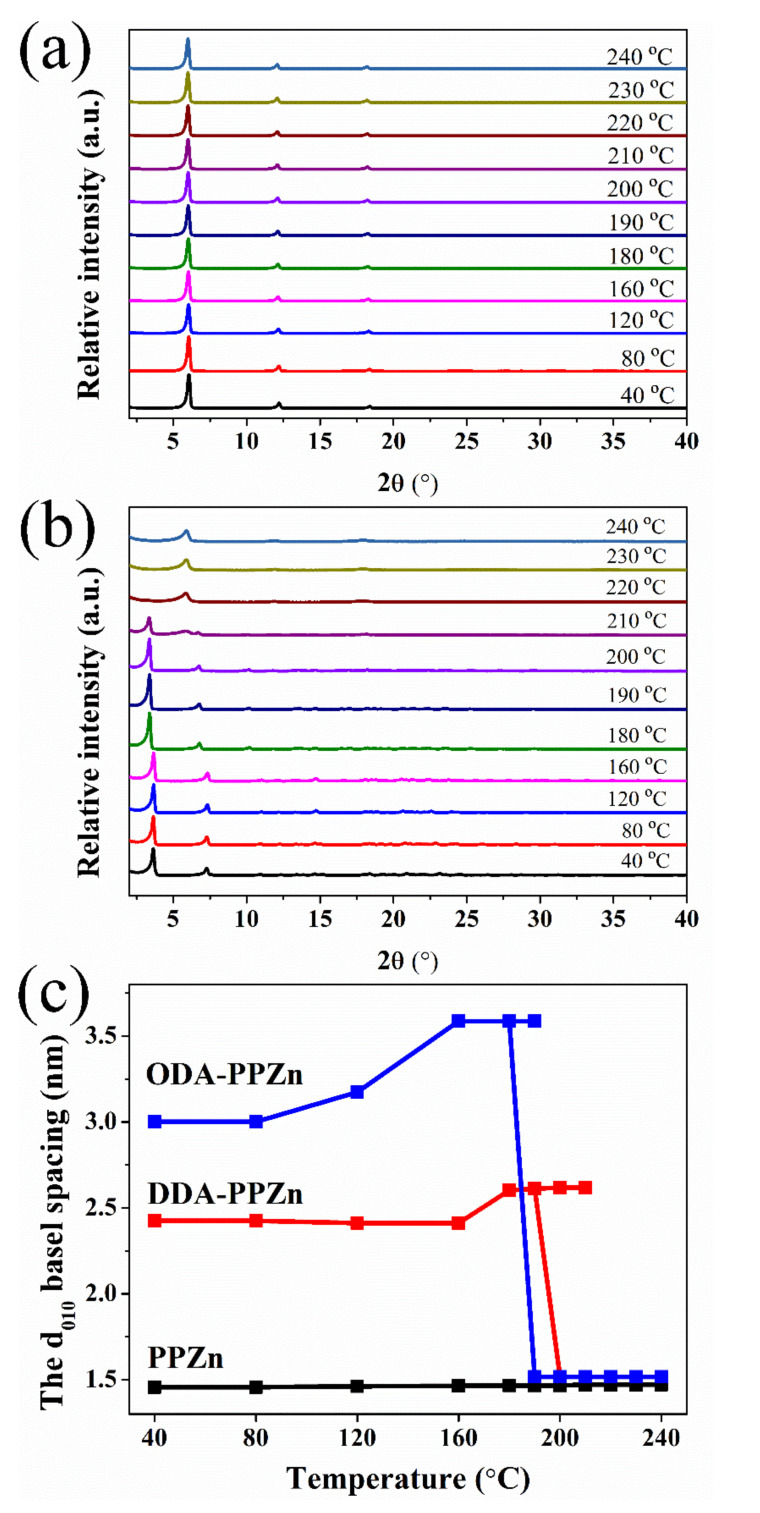
In situ wide-angle x-ray diffraction (WAXD) patterns of (**a**) PPZn and (**b**) DDA-PPZn in the temperature range of 40–240 °C. (**c**) The relationship between the d010 basal spacing and temperature of PPZn, DDA-PPZn, and ODA-PPZn.

In addition, in situ WAXD measurements helped us to understand the structure and behavior of DDA and ODA in the interlayer space of PPZn. Figure 3a,b present the in situ WAXD curves of PPZn and DDA-PPZn from 40 to 240 °C. It can be observed in Figure 3a that the strong (010) and (020) diffraction peaks remain almost the same with increasing temperature. This finding reveals that the interlayer spacing of PPZn is almost the same as the temperature increases. As observed for DDA-PPZn in Figure 3b, the (010) and (020) diffractions of m-PPZn that occurred at a lower 2*θ* angle remained almost the same from 80 °C to 160 °C. The in situ WAXD patterns of the (010) and (020) diffractions for DDA-PPZn slightly shifted to lower 2*θ* angle from 180 °C to 200 °C. This finding reveals that DDA-PPZn lattice is expanded with increasing temperature. At the temperature of 210 °C, the X-ray intensity of (010) plane is significantly decreased. At the same time, the diffraction peak of (010) plane for PPZn is observed. This result indicates that a mixture of DDA-PPZn and PPZN is present at this temperature, in which DDA is partially removed in the interlayer gallery of DDA-PPZn. However, the DDA was completely removed in the interlayer gallery of m-PPZn as the temperature reached 220 °C. The results recommend that the stacking structure of DDA-PPZn at 220 °C is almost the same as that of PPZn. Similar results are also observed for ODA-PPZn, except the temperature of structure change is decreased about 20–40 °C. To further examine the change of the interlayer distance of PPZn, DDA-PPZn, and ODA-PPZn, the d-spacings of the (010) diffraction calculated using Bragg’s equation for different temperatures are shown in Figure 3c. The interlayer distance of PPZn is almost the same as the temperature increases from 30 °C to 240 °C. In contrast, the d-spacings of the (010) diffraction for DDA-PPZn slightly increased from 160 °C to 200 °C and then significantly decreased from 26.2 to 14.7 Å as the temperature increased from 200 °C to 240 °C. At the same time, the interlayer distance of ODA-PPZn increased from 80 °C to 160 °C and then significantly decreased from 35.3 to 14.7 Å as the temperature increased from 190 °C to 200 °C.

**Figure 4:**
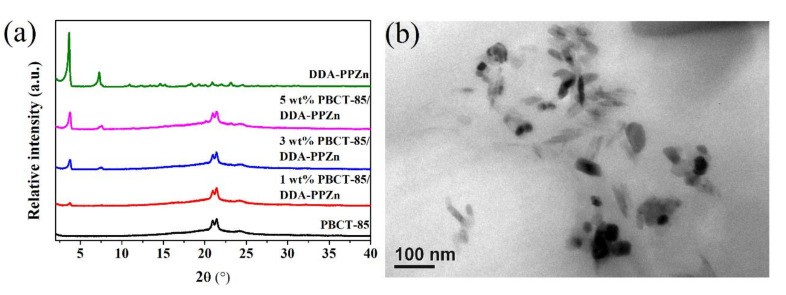
(**a**) WAXD patterns of PBCT-85 and various weight ratios of PBCT-85/DDA-PPZn nanocomposites. (**b**) TEM micrograph of 5 wt% PBCT-85/DDA-PPZn nanocomposites.

WAXD diffraction profiles of the PBCT-85/DDA-PPZn nanocomposites are exhibited in Figure 4a. For comparison, the X-ray diffraction data of DDA-PPZn is also presented in this figure. It is obvious that a clear sign of diffraction peak at 2*θ* = 3.64° was found in the experimental results of different loading of DDA-PPZn, which contributed to the presence of the stacking layers of the DDA-PPZn. These results recommend that the DDA-PPZn is well intercalated in the PBCT-85 copolymer matrix. Related results are also found for the PBCT-85/ODA-PPZn nanocomposites. Furthermore, the morphologies of 5 wt% PBCT-85/DDA-PPZn nanocomposites are wholly examined using TEM. Figure 4b presents the TEM image of 5 wt% loading of DDA-PPZn into PBCT-85 copolymer matrix. This image shows that the stacking layers of the DDA-PPZn are intercalated into the PBCT-85 copolymers. Related observations are also found for the PBCT-85/ODA-PPZn nanocomposites. Consequently, both WAXD and TEM experimental results of PBCT-85/m-PPZn nanocomposites recommend the presence of intercalated morphologies of nanocomposites. The degree of crystallinity (*Xc*) evaluated using WAXD data is shown in Table 2. These results show that the value of Xc of the PBCT-85/m-PPZn nanocomposites remained almost the same as the loadings of m-PPZn increase. This phenomenon contributed to the existence of m-PPZn in the PBCT-85 matrix, which probably caused almost similar effect on the heterogeneous nucleation and crystal growth rate, resulting in the similarity of the degree of crystallinity.

**Figure 5:**
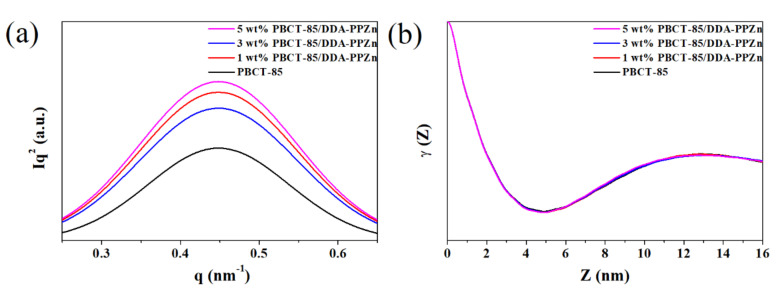
(**a**) Lorentz-corrected SAXS profiles and (**b**) one-dimensional correlation function calculated from the SAXS profiles for PBCT-85/DDA-PPZn nanocomposites.

The microstructures of PBCT-85/m-PPZn nanocomposites were examined by SAXS. The Lorentz-corrected SAXS data of PBCT-85/DDA-PPZn nanocomposites is shown in Figure 5a. To comprehensively investigate the structural parameters, such as long period (*L_p_*), the lamellar thickness (*l_c_*), and the amorphous thickness (*l_a_* = *L_p_* − *l_c_*), of the PBCT-85/DDA-PPZn nanocomposites, the Fourier transformation of Lorentz-corrected SAXS data were used to calculate the one-dimensional correlation function using the following equation [19,20]:

(1) $$\gamma (z)=\frac{1}{Q}\int_{0}^{\infty }qI^{2}(q)\cos(qz)dq$$

where *Q* is the invariant, *I*(*q*) is the Lorentz-corrected intensity, and *z* is the direction along which the electron density is measured.

The structural parameters of PBCT-85/DDA-PPZn nanocomposites are found from the typical profiles of one-dimensional correlation function presented in Figure 5b. Similar results are also obtained for the PBCT-85/ODA-PPZn nanocomposites. The obtained structural parameters of PBCT-85/m-PPZn nanocomposites are presented in Table 2. For the PBCT-85/DDA-PPZn nanocomposites, *L_p_* and *l_c_* remained almost the same with the DDA-PPZn content. This result indicates that the presence of stearic acid served as an intercalation agent for PPZn, which has few effects on the crystalline packing of PBCT-85 crystallites. Therefore, the calculated *l_a_*/*L_p_* and the degree of crystallinity of the nanocomposites determined using WAXD data remained unchanged regarding the loading of DDA-PPZn. For the PBCT-85/ODA-PPZn nanocomposites, *L_p_* and *l_c_* gradually increased with the ODA-PPZn content. Regarding the incorporation of ODA for PPZn intercalation, ODA-PPZn might enhance the migration of PBCT-85 chains to the packing of crystals. At the same time, the la values are also increased as the loadings of ODA-PPZn content increased. However, the calculated *l_a_*/*L_p_* and the degree of crystallinity determined using WAXD data remained unchanged as the loading of ODA-PPZn. These results suggest that the incorporation of m-PPZn into PBCT-85 polymer matrix did not change the microstructure of PBCT-85.

### 3.2. Physical Properties of PBCT/m-PPZn Nanocomposites

**Figure 6:**
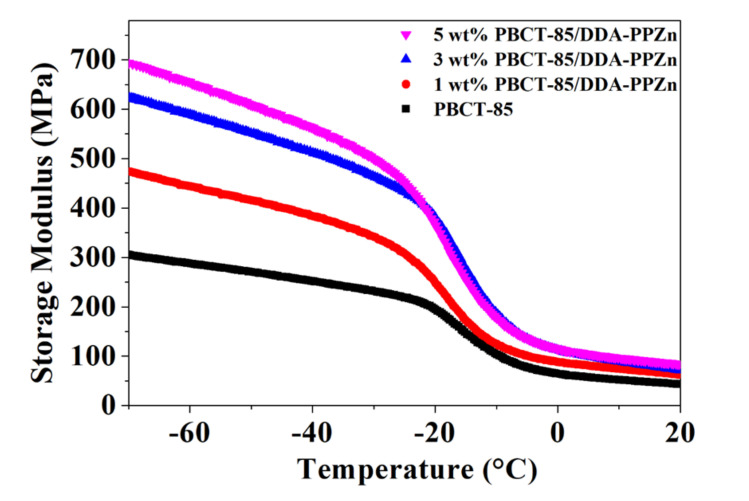
Dependence of the storage modulus on temperature of PBCT-85/DDA-PPZn nanocomposites.

Figure 6 shows the change of storage modulus E’ against temperature of PBCT-85/DDA-PPZn nanocomposites in a temperature range between −70 and 20 °C. These findings indicate that the E’ of PBCT-85 at −70 °C is around 305 MPa and decreases with an increasing temperature. This result suggests that the molecular movement of PBCT-85 in the region of glassy state is deficient. When the temperature higher than the glass transition temperature is increased, the thermal energy appears to be equivalent to the potential energy barriers of the molecular movements. The values of E’ of the PBCT-85/DDA-PPZn nanocomposites at −70 °C and 20 °C are increased as the loading of DDA-PPZn increases. Related results are also found for the PBCT-85/ODA-PPZn nanocomposites. Detailed E’ for all nanocomposites is also illustrated in Table 3. The improvement of E’ at −70 °C may be ascribed to the strengthening influence of the addition of the stiff and inorganic m-PPZn, causing the enhancement of the rigidity of the PBCT-85 polymer matrix. The change of E’ in rubbery state is not significant due to the increase in molecular chain movements. Similar phenomenon has been reported previously [21,22]. As the temperature reaches above the *Tg*, the effect of the PPZn incorporation on E′ becomes negligible and the rigidity of composites turns matrix-dependent.

**Figure 7:**
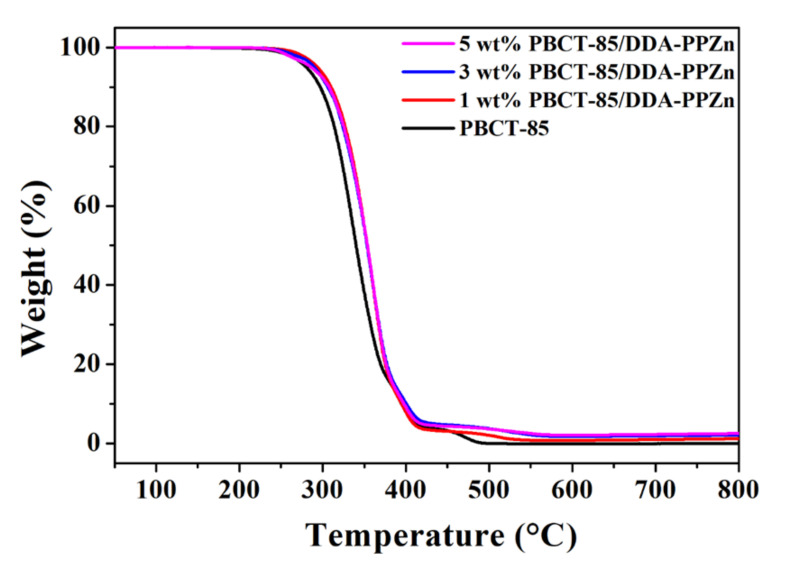
TGA curves of PBCT-85/DDA-PPZn nanocomposites.

The effect of m-PPZn on the thermal degradation of the various PBCT matrices has been studied using thermogravimetric analysis (TGA). Figure 7 reveals the TGA curves of the PBCT-85/DDA-PPZn nanocomposites. These profiles present the weight loss as the temperature increases; the degradation temperatures obtained from these profiles are reported in Table 3. As displayed in this table, the initial degradation temperature of the PBCT-85/m-PPZn nanocomposites increases compared to that of the pure PBCT-85 copolymer. This phenomenon is ascribed to fact that the presence of higher thermal stability of inorganic materials of m-PPZn in the PBCT-85 matrix induced better thermal stability, and thus the initial degradation temperature evidently moved into higher temperatures.

**Figure 8:**
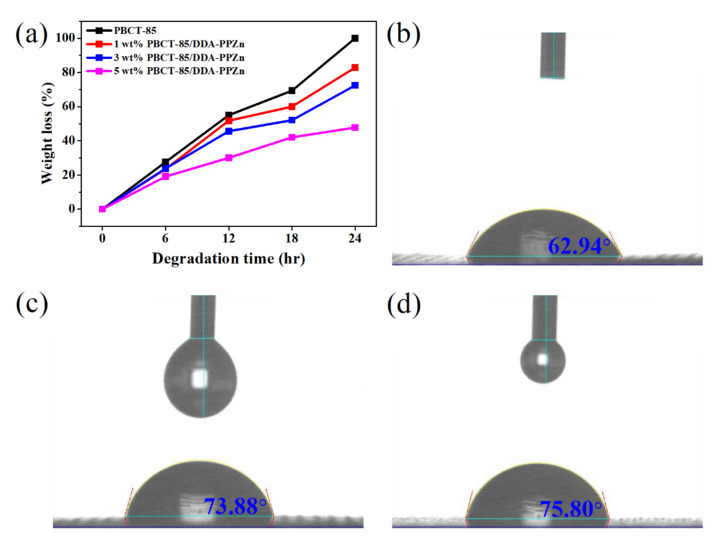
(**a**) Dependence of the weight loss on the degradation time for the PBCT-85/DDA-PPZn nanocomposites. Contact angles of (**b**) PBCT, (**c**) PBCT-85/DDA-PPZn, and (**d**) PBCT-85/ODA-PPZn nanocomposites.

For the enzymatic degradation test, the lipase from *Pseudomonas sp*. was utilized to study the effect of m-PPZn on the enzymatic degradation behavior of the PBCT-85/m-PPZn nanocomposites. Because m-PPZn cannot be degraded via lipase, the difference of weight loss after enzymatic degradation is related to the PBCT-85 copolymers. Figure 8a shows the weight losses of PBCT-85/DDA-PPZn nanocomposites after various degradation times. The weight loss of neat PBCT-85 after 24 h degradation was 100%; the weight losses for the PBCT-85/DDA-PPZn nanocomposites were 82.8%, 72.5%, and 47.8%, with loadings of 1, 3, and 5 wt% DDA-PPZn, respectively. The weight loss tendencies of PBCT-85/ODA-PPZn nanocomposites were similar to those of the PBCT-85/DDA-PPZn nanocomposites. Detailed weight loss after 24 h degradation for all nanocomposites is also illustrated in Table 3. The weight loss decreases as the incorporation of m-PPZn increases, recommending that the existence of m-PPZn inhibits the degradation of the PBCT-85 copolymers. Figure 8b shows the surface contact angle of PBCT-85, 5 wt% PBCT-85/DDA-PPZn, and 5 wt% PBCT-85/ODA-PPZn nanocomposites. This result shows that the contact angles of nanocomposites are relatively higher than of PBCT-85 polymer matrix. Higher contact angles lower hydrophilic properties. The change of weight loss of composites might have contributed to the lower hydrophilic properties for PBCT-85/m-PPZn nanocomposites.

**Figure 9:**
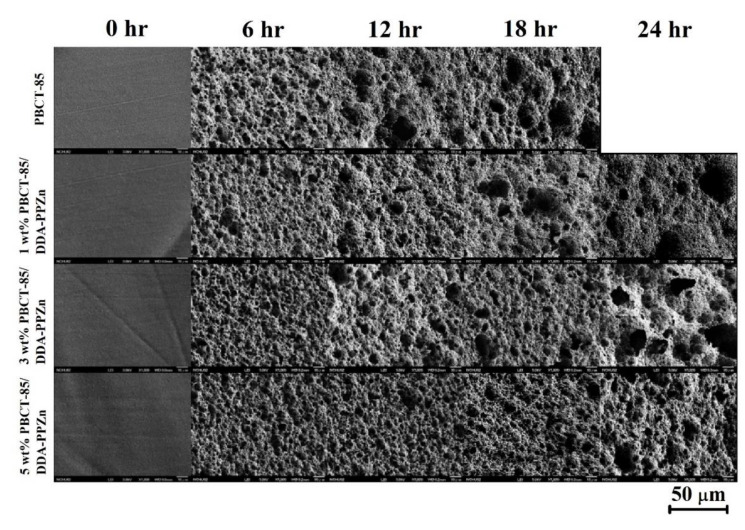
FESEM images of the enzymatically degraded PBCT-85/DDA-PPZn nanocomposites.

FESEM analysis can be used to examine the morphologies of all the nanocomposites after the enzymatic degradation. Figure 9 shows the FESEM images of the surfaces of all the nanocomposites before and after enzymatic degradation. Before enzymatic degradation, the surfaces of PBCT-85 and their nanocomposites were very smooth. After the degradation, the porous and holes structures were observed on the surface of PBCT-85 and all nanocomposites. It can be noticed that the surface roughness of the PBCT-85/m-PPZn nanocomposites decrease as the loadings of m-PPZn increase, recommending that the incorporation of the organically-modified PPZn content into PBCT significantly reduces the degradation rate of PBCT-85. Related results are also found for the PBCT-85/ODA-PPZn nanocomposites.

**Figure 10:**
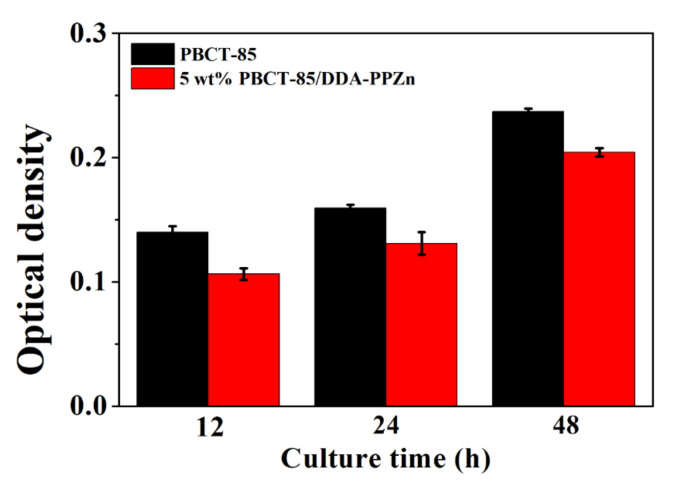
Biocompatibility of PBCT-85/DDA-PPZn seeded with L929 cells at various time points.

According to previous literature, there is no significant cytotoxicity for the layered zinc phenylphosphonates [17,18,23,24]. The were no related reports of the cytotoxicity of new synthesized PBCT-85 in the literature. In this investigation, two biocompatible materials were mixed to fabricate the PBCT-85/m-PPZn composites using solution mixing process. In the MTT assay, the optical density of purple formazan converted from the MTT can be used to represent the cell growth. The greater the absorbance, the larger number of living cells [25]. Figure 10 shows the quantitative results of the MTT assay for the PBCT-85/DDA-PPZn nanocomposites after incubation periods of 12, 24, and 48 h, respectively. The experimental results revealed that the number the living L929 cell in all samples increased as the culture times increased. This verified that the fabricated PBCT-85/m-PPZn composites were appropriate for cell growth and might be a potential candidate used as biomedical materials. Nevertheless, the fabricated composites might be considered ecologically-friendly materials that can be useful for environmental sustainability.

## 4. Conclusions

The new biodegradable and biocompatible PBCT-85/m-PPZn nanocomposites were fabricated using the transesterification and polycondensation process. WAXD and TEM results revealed that the intercalated conformations are formed for the PBCT-85/m-PPZn nanocomposites. The incorporation of m-PPZn into PBCT-85 matrix could enhance the storage modulus, as compared to that of neat PBCT-85. The weight loss decreases as the loading of m-PPZn increases, showing that the existence of m-PPZn inhibits the degradation of the PBCT-85 copolymers. This result might have contributed to the higher degree of contact angle for PBCT-85/m-PPZn nanocomposites. The results of the MTT assay show that the PBCT-85/m-PPZn composites may have a potential application for biomedical materials.

## Figures and Tables

**Table 1:** Composition and molecular weight of synthesized polyesters.

Polymer	Feed Ratio [DMC]/[DMT] (mol %)	Polymer Ratio ^a^ [DMC]/[DMT] (mol %)	*M_w_* (g/mol) × 10^4^	*M_n_* (g/mol) × 10^4^	PDI	T_m_ (°C)
PBCT-85	85/15	85.8:14.2	3.81	2.02	1.89	33.7

^a^ Composition measured by ^1^H-NMR.

**Table 2:** Structural parameters and crystallinity of PBCT-85/DDA-PPZn and PBCT-85/ODA-PPZn obtained by X-ray scattering (SAXS) and WAXD measurements.

Sample	*L_P_* (nm)	*l_c_* (nm)	*l_a_* (nm)	*l_a_*/*L_P_* (%)	*Xc* (%)
PBCT-85	12.80	2.72	10.08	78.73	39.5
1 wt% PBCT-85/DDA-PPZn	12.84	2.76	10.08	78.78	39.6
3 wt% PBCT-85/DDA-PPZn	12.80	2.73	10.07	78.70	39.4
5 wt% PBCT-85/DDA-PPZn	12.80	2.72	10.08	78.77	39.5
1 wt% PBCT-85/ODA-PPZn	13.01	2.77	10.24	78.71	39.7
3 wt% PBCT-85/ODA-PPZn	13.70	2.92	10.78	78.70	39.6
5 wt% PBCT-85/ODA-PPZn	13.80	2.94	10.86	78.70	39.7

**Table 3:** The temperature of the maximum degradation rate, storage modulus, and weight loss after 24 h degradation time of the various PBCT/m-PPZn nanocomposites.

Sample	*^a^T_d_^max^* (°C)	*E*′ at −70 °C (MPa)	*E*′ at 20 °C (MPa)	Weight Loss (%)
PBCT-85	340.1	306 ± 31	42 ± 10	100
1 wt% PBCT-85/DDA-PPZn	354.1	473 ± 25	62 ± 4	82.8
3 wt% PBCT-85/DDA-PPZn	354.4	624 ± 8	71 ± 4	72.5
5 wt% PBCT-85/DDA-PPZn	354.9	698 ± 4	83 ± 9	47.8
1 wt% PBCT-85/ODA-PPZn	354.3	441 ± 4	51 ± 4	52.0
3 wt% PBCT-85/ODA-PPZn	355.1	610 ± 12	60 ± 3	45.6
5 wt% PBCT-85/ODA-PPZn	355.5	684 ± 5	72 ± 6	30.2

^a^*T_d_^max^*: Temperature of the maximum degradation rate.
